# Ubiquitous expression of an activating mutation in the *Pik3ca* gene reprograms glucose and lipid metabolism in mice

**DOI:** 10.1371/journal.pone.0322544

**Published:** 2025-05-12

**Authors:** Sabrina Caiazzo, Matthew J. Watt, Garron T. Dodd, Jacqueline Bayliss, Helen Thomas, Lorey K. Smith, Camilla B. Mitchell, Wayne A. Phillips

**Affiliations:** 1 Department of Cancer Research, Peter MacCallum Cancer Centre, Melbourne, Victoria, Australia; 2 Sir Peter MacCallum Department of Oncology, The University of Melbourne, Parkville, Victoria, Australia; 3 Department of Anatomy and Physiology, School of Biomedical Sciences, Faculty of Medicine Dentistry and Health Sciences; The University of Melbourne, Parkville, Victoria, Australia; 4 Immunology and Diabetes Unit, St. Vincent’s Institute of Medical Research, Fitzroy, Victoria, Australia; 5 Department of Biochemistry and Molecular Biology, School of Biomedical Sciences, Monash University, Melbourne, Victoria, Australia; The University of Texas Health Science Center at Houston, UNITED STATES OF AMERICA

## Abstract

Mutations in *PIK3CA*, the gene encoding the p110α catalytic subunit of PI3K, are among the most common mutations in human cancers and overgrowth syndromes. The ubiquitous expression of the activating *Pik3ca*^*H1047R*^ mutation results in reduced survival, organomegaly, hypoglycaemia and hypoinsulinemia in mice. Here we demonstrate that *in vivo* expression of *Pik3ca*^*H1047R*^ attenuates the rise in blood glucose in response to oral glucose administration, stimulates glucose uptake in peripheral tissues, inhibits hepatic gluconeogenesis and pancreatic insulin secretion, and increases adipose lipolysis and white adipose tissue browning. Together, our data reveal that the systemic activation of the PI3K pathway in mice disrupts glucose homeostasis through the regulation of hepatic gluconeogenesis, and leads to increased lipolysis of adipose tissue.

## Introduction

Phosphoinositide 3-kinase (PI3K) mediated signalling is a critical intracellular signalling pathway, regulating a plethora of cellular functions including cell growth and proliferation, differentiation, motility, survival, angiogenesis, immunity, and metabolism [1–3].

PI3Ks are a group of heterodimeric lipid kinases that generate a range of intracellular lipid second messengers by phosphorylating phosphatidylinositol (PI) lipids at the 3-hydroxyl group of their inositol ring [4,5]. Class 1A PI3Ks are activated by receptor tyrosine kinases which recruit PI3Ks to the plasma membrane thereby activating the catalytic component of the PI3K and bringing it in close proximity to its membrane-associated substrates [6]. PI3K is able to phosphorylate PI to generate phosphatidylinositol‐3‐phosphate, phosphatidylinositol-4-phosphate to form phosphatidylinositol‐3,4‐bisphosphate, and phosphatidylinositol-4,5-bisphosphate (PIP_2_) to produce phosphatidylinositol‐3,4,5‐trisphosphate (PIP_3_) [7]. PIP_3_ activates a number of downstream targets including the protein kinase AKT [8].

The PI3K pathway is regulated by the phosphatase and tensin homolog, PTEN, a 3-phosphatase that terminates PI3K signalling by dephosphorylating PIP_3_ back to PIP_2_. PIP_3_ can also be dephosphorylated by 4- and 5-phosphatases to produce phosphatidylinositol-3,5-bisphosphate and phosphatidylinositol-3,4-bisphosphate, respectively [9].

A significant metabolic function of the PI3K pathway is the regulation of glucose homeostasis [10]. PI3K has been shown to play an essential role in controlling insulin secretion from the β-cells of the pancreas with deletion of Class IA PI3K decreasing β-cell mass and insulin production in mice [2]. Insulin exerts pleiotropic actions including stimulation of glucose uptake in peripheral tissues by promoting the PI3K-mediated trafficking of the glucose transporter 4 (GLUT4) to the membrane of insulin-sensitive tissues [11], restoring physiological levels of glucose in the blood. In addition to glucose uptake, the PI3K-AKT axis can both directly and indirectly regulate glycolysis through post-translational modification (e.g., phosphorylation, glycosylation) of metabolic enzymes [12,13]. It can also downregulate the expression of genes encoding key regulatory proteins of gluconeogenesis such as phosphoenolpyruvate carboxykinase (*PEPCK*) and glucose-6-phosphatase (*G6PC*) [14].

Insulin is also a powerful modulator of adipose tissue metabolism. It promotes *de novo* lipid biosynthesis by regulating the activity of SREBP and FOXO1 [15], and inhibits lipolysis through regulation of protein kinase A activity (PKA) [16]. In the fasting state PKA stimulates lipolysis through PKA-mediated phosphorylation of several lipases, including adipose triglyceride lipase (ATGL) [17] and hormone sensitive lipase [18], and interacting proteins such as perilipin 1 and 5 [19,20]. In the fed state, PI3K/AKT phosphorylates and activates phosphodiesterase 3b (PDE3b), which catalyzes the hydrolysis of cAMP to 5′AMP, thereby attenuating PKA activity and lipolysis [16].

Activating mutations in *PIK3CA*, the gene encoding the p110α catalytic subunit of PI3K, are among the most common mutations in human cancers [21]. They have also been shown to be responsible for various congenital overgrowth and vascular malformation syndromes [22,23]. In a previous study, we demonstrated that the ubiquitous expression of the common *Pik3ca* mutation, *Pik3ca*^*H1047R*^, results in increased body size and early lethality in mice [24]. Interestingly, the mice also presented with severe hypoglycaemia and hypoinsulinemia. Here we characterise the mechanism underlying such metabolic changes resulting from the ubiquitous activation of the PI3K pathway. We show that ubiquitous expression of the *Pik3ca*^*H1047R*^ mutation increases glucose uptake in peripheral tissues, blocks hepatic gluconeogenesis, and increases adipose tissue lipolysis and white adipose tissue (WAT) browning in mice. Interestingly, the *Pik3ca*^*H1047R*^ mutation was also found to prevent insulin release from pancreatic islet cells in response to glucose.

## Experimental procedures

### Mice

Mice harbouring a homozygous latent Cre recombinase (Cre)-inducible *Pik3ca*^*H1047R*^ mutation (*Pik3ca*^*lat-H1047R/lat-H1047R*^) [25] were crossed with mice heterozygous for a ubiquitously expressed tamoxifen-activatable Cre (*UbCreERT2*; Tg (UBC-cre/ESR1)1Ejb/J; The Jackson Laboratory, Bar Harbor, ME, USA) [26], to generate mice heterozygous for both the *Pik3ca*^*lat-H1047R*^ and the *UbCreERT2* alleles (*Pik3ca*^*lat-H1047R*^:*UbCreERT2*) and littermate Cre-negative control mice (*Pik3ca*^*lat-H1047R*^). To activate the Cre and knock-in the *Pik3ca*^*H1047R*^ mutation, tamoxifen (Sigma-Aldrich, St. Louis, MO) solubilised at 30 mg/mL in a mixture of 98% sunflower oil and 2% ethyl alcohol was administered by oral gavage using a 20 G x 33 mm flexible gavage needle at a dose of 200 mg/kg, on 2 consecutive days. This produced mice (from here on referred to as *Pik3ca*^*H1047R*^ mutant mice) that express the *Pik3ca*^*H1047R*^ mutation, and elevated PI3K pathway activity, in all cells of the body that normally express the endogenous *Pik3ca* gene [24]. All mice were maintained on a C57BL6 background and fed a standard chow diet (irradiated Barastoc mouse cubes; Ridley AgriProducts, Melbourne, Australia). Tamoxifen was administered at 6–8 weeks of age and all tests were performed 6 or 7 days post primary administration of tamoxifen.

All animal experiments were approved by the Peter MacCallum Cancer Centre Animal Ethics Committee (Project Number E584) and/or the University of Melbourne Animal Ethics Committee (ID No: 1914851.1), as appropriate, and conducted by appropriately trained staff in strict accordance with the Australian Code for the Care and Use of Animals for Scientific Purposes (the Code). Mice were monitored daily (grooming, coat condition and behavior changes) post tamoxifen administration and immediately and humanely euthanised at any sign of discomfort and/or distress (hunching, ruffled fur, lack of movement, reduced/excessive grooming, etc.) or a > 20% loss in body weight. All mice were humanely euthanised by cervical dislocation or carbon dioxide asphyxiation at the conclusion of the experiments.

### Immunohistochemistry

For inguinal WAT and interscapular brown adipose tissue (BAT) immunohistochemistry, animals were euthanised and tissues immediately dissected and fixed in buffered formalin solution for 24 hours. Tissues were embedded in paraffin and 4 μm sections of the entire block prepared. Every tenth to fourteenth section of the tissue was used to detect UCP-1 or tyrosine hydroxylase (TH) by immunohistochemistry. Slides were baked at 60°C for one hour in an environmental chamber (Labec Laboratory Equipment, NSW, AU), then dewaxed (Leica Biosystems, 82 Mount Waverley Victoria, AU). Sections were subjected to antigen retrieval in citrate acid buffer (pH 6.0) at 95°C for 20 minutes, then rinsed in water (5 minutes) and Tris-buffer saline containing 1% tween 20 (TBS-T) (5 minutes). Sections were then blocked with 3% (v/v) hydrogen peroxide (H_2_O_2_) in water. After washing with TBS-T for 5 minutes, sections were blocked with 10% (w/v) bovine serum albumin (BSA) in TBS-T for 1 hour. Slides were incubated overnight (4°C) with anti-UCP-1 (1:1000; ab10983, Abcam, San Francisco, CA) or anti-TH (1:500; AB152, Millipore, Billerica, MA). After washing for three times in TBS-T, sections were then incubated with an anti-rabbit DAKO envision + system-HRP labelled polymer (DAKO, Agilent Technologies Australia Pty Ltd, Mulgrave VIC, AU), again washed three times with TBS-T and then incubated for 1–2 minutes with DAKO liquid DAB+ substrate chromogen system (DAKO, Agilent Technologies Australia). Following a final wash with water for 5 minutes, the slides were counterstained with hematoxylin and eosin using the automatic slide stainer (Leica Biosystems, Mount Waverley Victoria, AU) and coverslipped (DAKO, Agilent Technologies Australia Pty Ltd). Images were acquired using the OLYMPUS VS120 slide scanner (Olympus Australia Pty Ltd, Notting Hill VIC, AU) and visualised using the OlyVIA software (Olympus Life Science) using 40X magnification.

### RT-PCR

Complementary DNA (cDNA) was synthesised from the RNA extracted from mice tissues by using the transcription first strand cDNA synthesis kit (Roche Diagnostics, Castle Hill, NSW, Australia). The cDNA was used as template for the q-RT-PCR. PCR was performed using SYBR Green qPCR Master Mix (LightCycler^®^ 480 SYBR green, Roche Diagnostics, Castle Hill, NSW, Australia) at the LightCycler^®^ 480 Instrument (Roche Diagnostics, Castle Hill, NSW, Australia). The following primers were used for q-RT-PCR assays: *Cd137* (f-CGTGCAGAACTCCTGTGATAAC, r-GTCCACCTATGCTGGAGAAGG), *Tmem26* (f-ACCCTGTCATCCCACAGAG, r-TGTTTGGTGGAGTCCTAAGGTC), Npy (f-CAAGAGCAACAACTCGGCATT, r-GAGAGGGACAGGTTGGCAATC), *Pomc* (f-ATGCCGAGATTCTGCTACAGT, r-TCCAGCGAGAGGTCGAGTTT), *Agrp* (f-ATGCTGACTGCAATGTTGCTG, r-CAGACTTAGACCTGGGAACTCT). Changes in gene expression were analysed using the ΔΔC_t_ method and normalised against *Actb* (f-CATGTACGTTGCTATCCAGGC, r-CTCCTTAATGTCACGCACGAT).

### Glucose and pyruvate tolerance tests

For glucose and pyruvate tolerance, mice were fasted for 3 hours before glucose (2 g/kg by oral gavage) or pyruvate (2 g/kg by intraperitoneal injection) administration. Blood glucose levels were measured using a hand-held glucometer (Accu-Check Performa; Roche Diagnostics, Castle Hill, NSW, Australia) after 0, 15, 30, 45, 60, 90 and 120 minutes. Additional blood was collected during the glucose tolerance test at 0, 15 and 30 min to assess plasma insulin levels using a mouse Insulin ELISA kit (Ultra-sensitive Insulin ELISA, Crystal Chem, Elk Grove Village, IL, USA).

### Tracer [^18^F] Fluorodeoxyglucose (FDG) biodistribution

Mice were fasted for 3 hours before being anaesthetised with a mixture of 2–4% isoflurane and 1 L/min 100% oxygen. ^18^F-FDG (Cyclotek, Bundoora, VIC, AU) (~2–3 MBq per mouse) was injected intravenously (i.v.) in the tail of the mice. One hour after the injection, mice were sacrificed by cervical dislocation. Blood was then collected through cardiac puncture, and brain, muscle, small and large intestine, pancreas, liver, lung, spleen, heart and kidney were harvested and weighed. The radioactivity present in each tissue was measured using a γ-counter (PerkinElmer, Melbourne, VIC, AU) and expressed as CPM/tissue weight. Results were adjusted on the time of injection and on the decay activity of the original injection solution and listed as a percentage of the injected dose per gram of tissue mass (% ID/g).

### Glucose stimulated insulin secretion

Mouse islets of Langerhans were isolated using collagenase P (Roche, Basel, Switzerland) and Histopaque-1077 density gradients (Sigma-Aldrich) as previously described [27]. Pancreatic islets were washed 3 times in a petri dish with Krebs-Ringer Modified Buffer (KRB) (460 mM NaCl, 20 mM KCl, 95.5 mM NaHCO_3_, 4 mM MgCl_2_.6H_2_O, 10 mM CaCl_2_ dehydrate, 5 ml 0.5 M HEPES, 20 ml dH_2_O, 54 mg D-glucose (final concentration of 3 mM). Twenty islets were added to siliconized glass tubes. Each tube was gassed with 95% air/5% CO_2_ and incubated for 30 minutes at 37°C on a shaker. KRB (3mM glucose) was removed and replaced with 200 µl of KRB containing either 3 mM or 20 mM glucose. The supernatant was collected and the insulin level assessed using a commercially available mouse insulin enzyme-linked immunoassay (ELISA) kit (Mercodia, Sapphire Bioscience, Redfern NSW 2016, AU), following the instructions of the manufacturer.

### Plasma lactate analysis

Plasma lactate was measured using the L-Lactate assay kit (Jomar Life Research, Caribbean Park, Australia) following the manufacturer’s instructions. A standard curve was prepared by adding 0.3–2 mM lactate standards in duplicate. Lactate reagent (15 μl) (Jomar Life Research) was add to each well of a 96 wells plate and incubated in a CO_2_-free incubator at 37°C for 45 minutes. Acetic acid (0.5 mM) was add to each well (15 μl per well) to stop the reaction. Absorbance was read at 490nm (A_490_) using the Cytation 3 Cell Imaging Multi-Mode Reader (Millennium Science Australia Pty Ltd, Mulgrave, Australia). Plasma lactate concentration was interpolated using the standard curve and mean absorbance values for each sample.

### Metabolic cage analysis

Mice were transferred to the University of Melbourne Biomedical Science Animal Facility (BSAF, Parkville, Victoria 3010, AU) and individually housed into Promethion cages (Promethion®, Sable Systems, LasVegas, NV), for a period of 2.5 days. The first 12 hours in the metabolic cages were necessary for acclimation of the mouse to the new cage, and the subsequent hours were used to obtain stable values. Mice were monitored daily for signs of distress, abnormal feeding and drinking behaviour, and changes in body mass. Water bottles and food hoppers were connected to load cells for food and water intake monitoring. Ambulatory activity and position of the mice in the cage were detected with XYZ beam arrays (BXYZ-R, Sable Systems, LasVegas, NV). Integrated fuel cell oxygen analyser, spectrophotometric CO_2_ analyser and capacitive water vapour partial pressure analyser (GA3, Sable Systems, Las Vegas, NV) were connected to the cages to measure oxygen consumption and CO_2_ production for each mouse at 5 minute intervals (Kaiyala et al., 2012). The RER was calculated as the ratio of CO_2_ production over O_2_ consumption. Energy expenditure (EE) measures were obtained using a computer controlled indirect calorimetry system (Promethion®, Sable Systems, LasVegas, NV). The EE Analysis of Covariance (ANCOVA) analysis was informed by the NIDDK Mouse Metabolic Phenotyping Centers (MMPC, www.mmpc.org) Energy Expenditure Analysis guide (http://www.mmpc.org/shared/regression.aspx).

### Body composition analysis

Fat mass, lean mass and free fluid of mice were measured by Time Domain Nuclear Magnetic Resonance (TD-NMR), using a MiniSpec whole body composition analyser (LF50, Bruker Analyzer, Billerica, Massachusetts, United States). Once the scan was complete the restrainer was removed and mice were returned to their original cages. Data were expressed as percentage of fat, lean or fluids normalised to the body weight of the mice.

### Lipid metabolism measurements

For the analysis of lipolysis, epididymal fat and periovarial fat were incubated in phenol red-free low glucose DMEM (Dulbecco’s Modified Eagle Medium, Sigma-Aldrich, Castle Hill NSW 2154, AU) containing 2% BSA, and 1) no additions (= basal lipolysis), 2) 1 μM isoproterenol (β-adrenergic stimulation), or 3) 20 μM forskolin (activates protein kinase A by elevating cAMP levels), at 37°C in a shaking water bath for 2 hours. Adipose tissue was rinsed in saline solution (0.9% NaCl), frozen in liquid nitrogen and stored at -80°C. Glycerol released into the incubation medium was measured using the free glycerol reagent according to the manufactuer’s instructions (Sigma Aldrich, Castle Hill NSW 2154, AU).

### Fatty acid oxidation measurement

For the analysis of fatty acid oxidation, mice were fasted for 2–4 hours and then sacrificed with an intraperitoneal injection of pentobarbital sodium. The soleus muscle was dissected tendon to tendon, then an approximately 20 mg portion of liver was excised using a surgical blade. Tissues were placed in glass vials containing 2 ml of warmed (30°C), pre-gassed (Carbogen: 95% O_2_/5% CO_2_,), DMEM (pH 7.4) containing 1% BSA, 100 mM carnitine, 100 mM oleate and 1 μCi/ml ^14^C-labelled oleate. Tissues were incubated for 2h, then rinsed in saline solution (0.9% NaCl), frozen in liquid nitrogen and stored at -80°C until further analysis. Tissues were processed as previously described [20]. Briefly, fatty acid oxidation was calculated by the production of ^14^CO_2_ (complete oxidation) and accumulation of ^14^C in an acid-soluble metabolite fraction (ASM, incomplete oxidation). Incorporation of [1-^14^C] oleate into endogenous lipids was assessed by thin-layer chromatography. Fatty acid uptake was calculated by adding total oxidation to total fatty acid storage. Radioactivity was detected using a scintillation counter (Tri-Carb 4910TR liquid scintillation analyser (PerkinElmer, Melbourne, Victoria 3150, AU)).

### Sympathetic denervation

Mice were anesthetised with isofluorane and the right epididymal pad in each mouse was injected 20 times with 1 μl injections of 9 mg/ml 6-hydroxydopamine (6-OHDA) in 0.15 M NaCl containing 1% (w/v) ascorbic acid. Injections were given using a micro-syringe fitted with a 30-gauge needle. The needle was held in place for 45 seconds after each injection to minimise backflow. Sham-operated fat pads received an equal volume of vehicle. Two weeks after 6-OHDA injections, mice were euthanised and inguinal WAT and BAT were either formalin-fixed for histological/immunohistochemical assessment or processed for quantitative real time PCR.

### Hepatic glucose output

Mice were euthanised and their liver lobes removed. The liver was cut into 300 μm slices using a Krumdieck Tissue Slicer (Alabama Research & Development, Munford, AL). Slices were incubated in Medium 199 for 1 hour and then washed in phosphate-buffered saline. Slices were incubated in low glucose (0.9–1.1 g/l) DMEM supplemented with 10 mM lactate and 10 mM pyruvate with or without insulin (10 nM). The medium was collected after one hour, and the glucose levels were calculated using an enzymatic spectrophotometric method (Infinity^TM^ Glucose Oxidase Liquid Stable Reagent (Thermo Scientific). Glucose output was normalised to liver weight.

### Statistical analysis

Data were analysed using Microsoft® Office Excel (Microsoft, Redmond, WA, USA) and Prism-GraphPad (GraphPad software, San Diego, CA). Statistical significance was determined using a t-test or two-way ANOVA, with Bonferroni post hoc tests for multiple comparisons, as appropriate. p < 0.05 was considered significant.

## Results

### Expression of *Pik3ca*^*H1047R*^ leads to hypoglycaemia in mice

**Fig 1:**
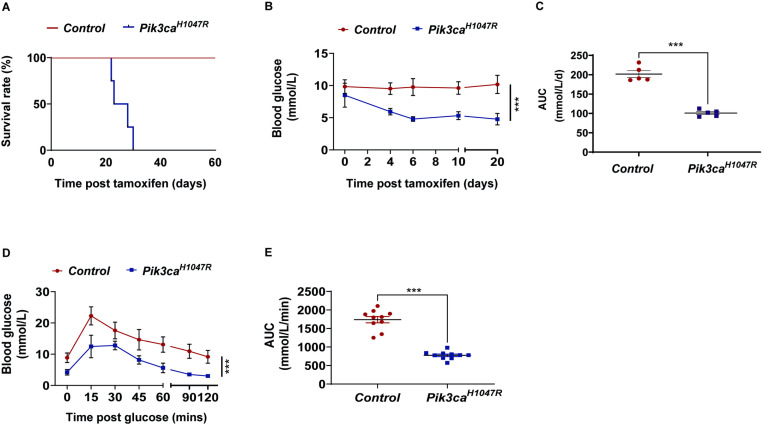
Survival rate and glucose metabolism in *Pik3ca*^*H1047R*^ mice. **(A)** Kaplan-Meier survival curve of control (*Pik3ca* wild type) and *Pik3ca*^*H1047R*^ mutant mice following induction with tamoxifen (tam; 200 mg/kg on 2 consecutive days) (n = 5 control, 5 *Pik3ca*^*H1047R*^). Blood glucose levels were measured regularly for 20 days and expressed as **(B)** absolute blood levels (mmol/L) and **(C)** area under the curve (AUC) (mmol/L/day) (n = 5 controls and 5 *Pik3ca*^*H1047R*^ mice). **(D-E)** Blood glucose levels following a bolus of glucose (2 g/kg by oral gavage) delivered 7 days post tamoxifen administration and expressed as **(D)** absolute blood levels (mmol/L) and **(E)** as area under the curve (AUC), expressed in mmol/L/min (n = 10 controls and 10 *Pik3ca*^*H1047R*^ mice). Data for **(B)** and **(D)** shown as mean ± SEM. Statistical significance was determined using two-way ANOVA with Bonferroni post hoc tests. *** p < 0.001. For **(C)** and **(E)**, each data point represents an individual mouse. Bars represent mean ± SEM. Statistical significance was determined by t-test. *** p < 0.001.

Activation of the *Pik3ca*^*H1047R*^ mutation with tamoxifen reduced the median survival of the mice to 25.5 days post tamoxifen treatment, compared to Cre-negative (i.e., *Pik3ca*^*wt*^) control mice, which were euthanised 60 days post drug treatment with no pathologic phenotype (Fig 1A). *Pik3ca*^*H1047R*^ mutant mice showed early and severe hypoglycaemia within 4 days of tamoxifen treatment (Fig 1B-C), and improved glycaemic control as assessed during an oral glucose tolerance test compared with control mice (Fig 1D-E). These results confirm previous findings of hypoglycaemia in *Pik3ca*^*H1047R*^ mice [24].

### Ubiquitous activation of the PI3K pathway stimulates glucose uptake from peripheral tissues

**Fig 2:**
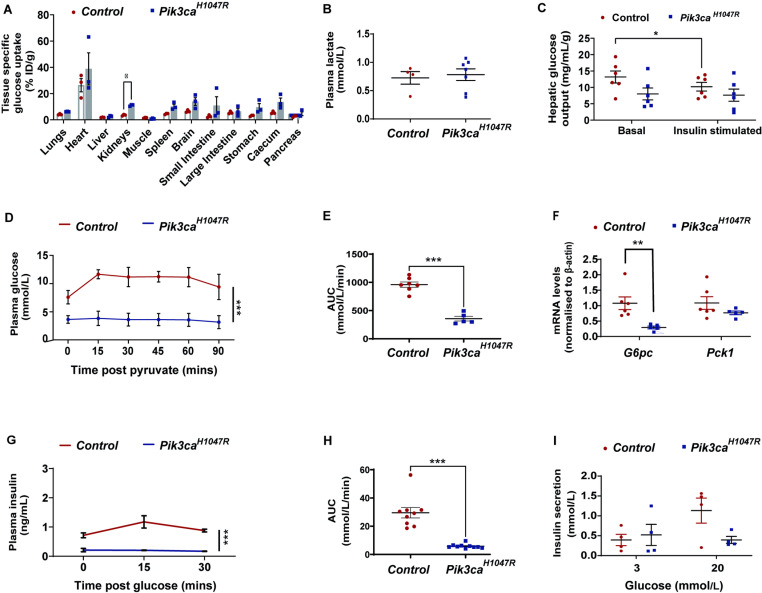
Pancreatic and hepatic regulation of glucose metabolism in *Pik3ca*^*H1047R*^ mice. Control and *Pik3ca*^*H1047R*^ mutant mice were assessed 7 days following administration of tamoxifen (200 mg/kg by oral gavage on two consecutive days). **(A)** ^18^F-FDG bio-distribution in mice tissues. Mice were injected with ~2-3 MBq radioactive ^18^F-FDG intravenously into the mouse tail vein. Mice were sacrificed 1 h post-injection of ^18^F-FDG and radioactivity assessed in individual tissues and expressed as the percentage of the injected dose per gram of tissue mass (% ID/g) (n = 3 controls and 3 *Pik3ca*^*H1047R*^ mice). **(B)** Lactate concentration in the plasma of *Pik3ca*^*H1047R*^ and control mice 7 days post tamoxifen administration (n = 4 controls and 7 *Pik3ca*^*H1047R*^ mice). **(C)** Production of glucose by cultured liver slices before (basal) and after (stimulated) *ex vivo* treatment with insulin (10 nM) (n = 6 controls and 6 *Pik3ca*^*H1047R*^ mice). **(D-E)** Pyruvate tolerance test. Mice were fasted for 3 hours prior to administration of pyruvate (2 g/kg body weight) by i.p. injection. Blood glucose was measured 0, 15, 30, 45, 60 and 90 minutes post injection of pyruvate and expressed as **(D)** absolute blood levels (mmol/L) and **(E)** area under the curve (AUC) (mmol/L/min) (n = 7 controls and 5 *Pik3ca*^*H1047R*^ mice). **(F)** Liver mRNA expression of the glucose-6-phosphatase (*G6pc*) and phosphoenolpyruvate carboxykinase (*Pck1*) in control and *Pik3ca*^*H1047R*^ mice, 7 days post tamoxifen administration, expressed as fold change relative to ß-actin (n = 6 controls and 5 *Pik3ca*^*H1047R*^ mice). **(G-H)** Plasma insulin levels following a bolus of glucose (2 g/kg by oral gavage) delivered 7 days post tamoxifen administration expressed as **(G**) absolute blood levels (ng/mL) and **(H)** area under the curve (AUC), (ng/mL/min) (n = 9 controls and 10 *Pik3ca*^*H1047R*^ mice). **(I)** Insulin release by isolated pancreatic islets cultured in the presence of 3 or 20 mM glucose. Mice were administered tamoxifen (200 mg/kg) by oral gavage, on two consecutive days, 7 days before harvesting the islets (n = 4 control, 4 *Pik3ca*^*H1047R*^). For **D** and **G** data are shown as mean ± SEM. For all other panels, each data point represents an individual mouse and bars represent mean ± SEM. Statistical significance was determined by t-test with Bonferroni correction for multiple comparisons **(A)**, two-way ANOVA with Bonferroni post hoc tests **(D and G)**, or standard unpaired t-test (all other panels). * p < 0.05, *** p < 0.001.

Having confirmed that ubiquitous expression of *Pik3ca*^*H1047R*^ leads to severe hypoglycaemia, we next sought to determine the underlying process. Insulin-mediated PI3K signalling is known to regulate glucose homeostasis by enhancing glucose disposal. Therefore, we primarily investigated whether the expression of the *Pik3ca*^*H1047R*^ mutation enhanced glucose uptake by the peripheral tissues by administering the glucose tracer [^18^F]-fluorodeoxyglucose (^18^F-FDG). The ^18^F-FDG was injected intravenously in the tail vein (~2–3 MBq per mouse) of the animals, 7 days post tamoxifen administration, a time at which blood glucose levels were stably reduced in *Pik3ca*^*H1047R*^ mice (Fig 1B). Compared to control mice, *Pik3ca*^*H1047R*^ mutant mice exhibited an enhanced uptake of labelled glucose in the kidneys, with a trend toward uptake also observed in most other tissues (Fig 2A).

Highly proliferative cells increase their dependence on glucose in order to fuel the production of cellular metabolites required for the generation of new biomass [28,29]. As our results suggest a trend towards an increased glucose uptake by the tissues, and since we had previously shown that *Pik3ca*^*H1047R*^ mutations increased tissue size [24], we next sought to investigate whether mice harbouring the *Pik3ca*^*H1047R*^ mutation have increased glycolysis. We indirectly assessed glycolysis by measuring the levels of the glycolytic product lactate in the plasma of control and *Pik3ca*^*H1047R*^ mice. No significant changes were observed in the plasma lactate of *Pik3ca*^*H1047R*^ mutant mice compared to control mice (Fig 2B) suggesting that glycolysis was not the reason for the observed hypoglycaemia in *Pik3ca*^*H1047R*^ mutant mice.

### Ubiquitous activation of the PI3K pathway inhibits gluconeogenesis

An especially important organ in controlling glucose homeostasis is the liver due to its ability to produce glucose, store glucose as glycogen, or utilise glucose for ATP production [30]. Activation of the insulin-PI3K pathway leads to inhibition of gluconeogenesis and glycogenolysis in the liver, decreasing glucose production and its release into the circulation [30]. We investigated hepatic glucose output from liver slices procured from control and *Pik3ca*^*H1047R*^ mutant mice. Liver slices from control mice responded to insulin stimulation *ex vivo* by reducing hepatic glucose output, whereas liver slices from *Pik3ca*^*H1047R*^ mice exhibited lower basal hepatic glucose output compared with control mice and were unresponsive to further insulin-mediated suppression of hepatic glucose output (HGO) (Fig 2C). To address whether *Pik3ca*^*H1047R*^ mice had defective hepatic gluconeogenesis *in vivo*, the metabolism of pyruvate (a substrate for hepatic gluconeogenesis) to glucose was measured using a pyruvate tolerance test (2 g/kg, i.p.). Whilst control mice were able to metabolise pyruvate to glucose, as shown by the increase in blood glucose within 15 minutes post injection of pyruvate, this was completely abrogated in *Pik3ca*^*H1047R*^ mutant mice (Fig 2D-E). Indeed, the blood glucose of *Pik3ca*^*H1047R*^ mice remained at baseline (less than 5 mmol/L) for the duration of the pyruvate challenge. Consistent with impaired hepatic gluconeogenesis, the hepatic expression of both glucose-6-phosphatase (*G6pc*) and phosphoenolpyruvate carboxykinase (*Pck1*), two rate-limiting enzymes involved in the synthesis of glucose, was reduced in *Pik3ca*^*H1047R*^ mice, compared to control mice (Fig 2F).

### Expression of *Pik3ca*^*H1047R*^ inhibits pancreatic release of insulin

To investigate whether mutations in the PI3K pathway affect glucose-stimulated insulin secretion, we administered glucose (2 g/kg) orally to the mice and measured the concentration of plasma insulin 0, 15 and 30 minutes post gavage of glucose. While the expected transient increase in blood insulin levels was observed in wild type mice, glucose-stimulated insulin release was completely absent in *Pik3ca*^*H1047R*^ mice (Fig 2G-H). These results suggest that the *Pik3ca*^*H1047R*^ mutation caused a dysfunction, either directly or indirectly, in pancreatic insulin secretion. To test this hypothesis, we isolated pancreatic islets from *Pik3ca*^*H1047R*^ mutant mice and wild type control mice and investigated glucose-stimulated insulin secretion *ex vivo* (Fig 2I). As expected, the addition of high glucose (20 mM) to the incubation medium resulted in a significant increase in insulin secretion, compared to low glucose, from islets isolated from control mice. In contrast, *Pik3ca*^*H1047R*^ mutant islets were unable to increase insulin secretion following exposure to high glucose. Thus, the expression of a heterozygous *Pik3ca*^*H1047R*^ mutation inhibits glucose–induced insulin release from pancreatic islets.

### Expression of *Pik3ca*^*H1047R*^ alters whole-body energy expenditure

**Fig 3:**
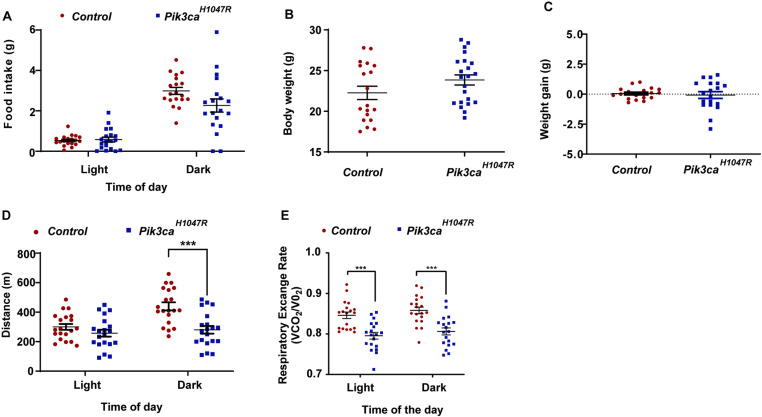
Food intake, and pedestrian activity in *Pik3ca*^*H1047R*^ mice. Control and *Pik3ca*^*H1047R*^ mutant mice were administered tamoxifen (200 mg/kg) by oral gavage on two consecutive days and 7 days later placed in Promethion cages. **(A)** Food intake was assessed in Promethion cages and expressed as the average weight (g) during the day (light) and night (dark) phases (n = 19 controls and 22 *Pik3ca*^*H1047R*^ mice). Total body weight was measured before and after Promethion cages and expressed as **(B)** absolute body weight (g) when first placed in the Promethion cage and **(C)** body weight gain (g) during the time in the cage (n = 19 controls and 22 *Pik3ca*^*H1047R*^ mice) **(D)**. Pedestrian activity was expressed as the average distance (m) covered during the day (light) and night (dark) phases. Each data point represents an individual mouse and bars represent mean ± SEM. Statistical significance was determined by t-test. *** p < 0.001.

We next addressed whether hypoglycaemia in *Pik3ca*^*H1047R*^ mutant mice was associated with changes in energy balance or increased carbohydrate oxidation. *Pik3ca*^*H1047R*^ mutant mice exhibited normal feeding behaviour (Fig 3A). Total body weight was similar between control and *Pik3ca*^*H1047R*^ mutant mice (Fig 3B) and no significant difference was observed in body weight gain between control and *Pik3ca*^*H1047R*^ mutant mice during the period of observation (Fig 3C) suggesting that the difference in blood glucose levels observed between control and *Pik3ca*^*H1047R*^ mutant mice was not due to changes in body weight. The physical activity of *Pik3ca*^*H1047R*^ mutant mice was reduced compared to control mice (Fig 3D) suggesting that the loss in blood glucose was not due to increased glucose consumption by the skeletal muscle during exercise. In addition, carbohydrate oxidation was actually reduced in *Pik3ca*^*H1047R*^ mutant mice compared with control mice, as indicated by a lower RER (Fig 3E).

**Fig 4:**
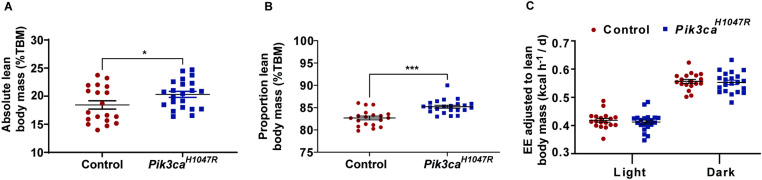
Energy expenditure in *Pik3ca*^*H1047R*^ mice. Whole lean body mass of mice was obtained by TD-NMR (assessed 6 days post-tamoxifen) and expressed **(A)** as absolute lean body mass (g) and **(B)** as percentage of total body weight (g) (n = 19 controls and 22 *Pik3ca*^*H1047R*^ mice). **(C)** Energy expenditure (EE) assessed in Promethion metabolic cages 7 days post-tamoxifen administration and expressed as EE adjusted lean body mass (kCal/h) during the day (light) and night (dark) phases. Each data point represents an individual mouse and bars represent mean ± SEM. Statistical significance was determined by ANCOVA. * p < 0.05 and *** p < 0.001.

Whole-body energy expenditure of control and *Pik3ca*^*H1047R*^ mutant mice was measured using indirect calorimetry. Interestingly, while the total lean mass of *Pik3ca*^*H1047R*^ mutant mice was increased compared to control mice (Fig 4A-B), overall energy expenditure was unaltered when ANCOVA-corrected for lean mass (Fig 4C). This suggests that the *Pik3ca*^*H1047R*^ mutation induces storage of energy into the lean mass to sustain growth.

### Ubiquitous activation of the PI3K pathway stimulates WAT browning

**Fig 5:**
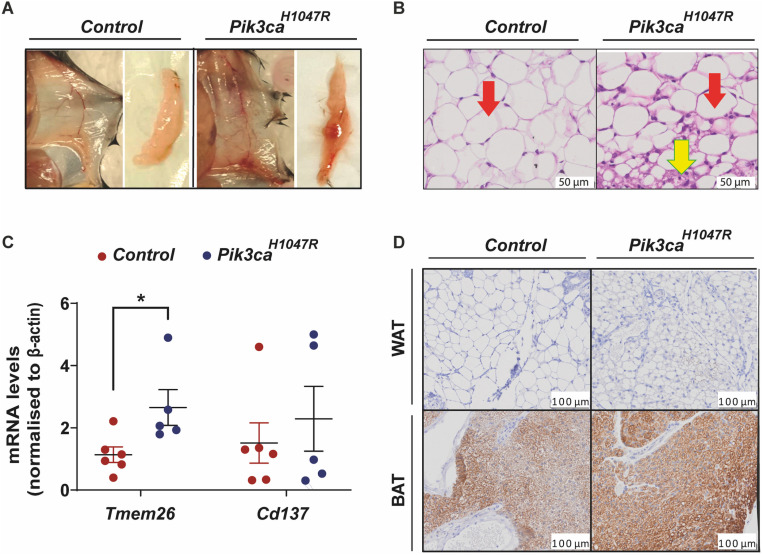
White adipose tissue browning in *Pik3ca*^*H1047R*^ mice. Control and *Pik3ca*^*H1047R*^ mutant mice were administered tamoxifen (200 mg/kg) by oral gavage on two consecutive days and euthanised for assessment 7 days post tamoxifen. **(A)** Gross morphology and **(B)** hematoxylin and eosin (H&E) staining of the inguinal fat pad. Red arrow indicates white adipocytes, yellow arrow indicates beige adipocytes. **(C)** mRNA levels of *Tmem26* and *Cd137* in the white adipose tissue (WAT), expressed as fold change relative to ß-actin. Each data point represents an individual mouse and bars represent mean ± SEM (n = 6 controls and 5 *Pik3ca*^*H1047R*^ mice). **(D)** UCP1 protein expression in the white adipose tissue (WAT) and brown adipose tissue (BAT) detected by immunohistochemistry. Statistical significance was determined by t-test. * p ≤ 0.05.

Glucose is metabolised in the white adipose tissue (WAT) to support the thermogenic capacity of beige adipocytes [31–33]. Beige adipocytes are found in various WAT deposits of mice, especially in the subcutaneous inguinal WAT. Similar to brown adipocytes in discrete regions of brown adipose tissue (BAT), beige adipocytes have many mitochondria, numerous smaller lipid droplets, and express uncoupling protein 1 (UCP1), which plays a pivotal role in thermogenesis [33]. UCP1 is activated through stimulation of the sympathetic nervous system and releases energy as heat. Previous studies have shown that the insulin-PI3K axis stimulates the activation and development of beige adipocytes, thus increasing energy expenditure and reducing obesity [34]. Thus, we explored whether ubiquitous expression of the *Pik3ca*^*H1047R*^ mutation affected WAT browning. The inguinal fat pad of *Pik3ca*^*H1047R*^ mice had a distinct gross morphology (Fig 5A) associated with dark brown colouration, and histological analysis demonstrated the presence of small clusters of adipocytes with a multilocular lipid droplet morphology typical of beige adipocytes [35] (Fig 5B). The presence of beige adipocytes was confirmed by increased expression of genes specific for beige adipocytes, *Tmem26* and *Cd137*, in WAT of *Pik3ca*^*H1047R*^ mice, relative to wild type control mice (Fig 5C). Multilocular beige adipocytes observed in the WAT were predominantly UCP1 negative (Fig 5D) whereas UCP1 expression in brown adipose tissue (BAT) was comparable between genotypes. This data suggests that *Pik3ca*^*H1047R*^ mutation uncouples respiration through an alternative mechanism that is potentially UCP1 independent. Recently, UCP1-independent thermogenic pathways have been identified in thermogenic adipocytes [36]. However, such mechanisms have not been investigated in this study.

**Fig 6:**
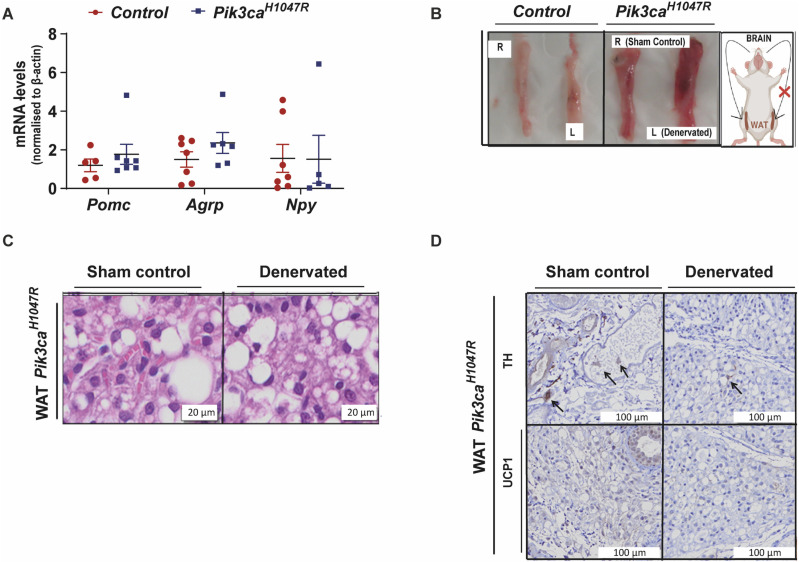
Hypothalamic effect on the white adipose tissue browning in *Pik3ca*^*H1047R*^ mice. Control and *Pik3ca*^*H1047R*^ mutant mice were administered tamoxifen (200 mg/kg) by oral gavage on two consecutive days. **(A)** mRNA expression of the *Pomc*, *Agrp*, *Npy* in the hypothalamus (n = 5-7 controls and 5-7 *Pik3ca*^*H1047R*^ mice), expressed as fold change relative to ß-actin. Each data point represents an individual mouse and bars represent mean ± SEM. **(B)** Gross morphology of representative contralateral sham control (R) versus denervated (L) inguinal fat pads from control and *Pik3ca*^H1047R^ mice. **(C)** Hematoxylin and eosin (H&E) staining of WAT in representative contralateral sham control and denervated inguinal fat pads from *Pik3ca*^H1047R^ mice. **(D)** TH and UCP1 staining of WAT in the contralateral sham control and denervated inguinal fat pads from *Pik3ca*^*H1047R*^ mice. Each data point represents an individual mouse and bars represent mean ± SEM.

Previous studies have established that the PI3K pathway regulates WAT browning by activating pro-opiomelanocortin (POMC) and suppressing the agouti-related neuropeptide (AgRP) expressing neurons in the arcuate nucleus of the hypothalamus [34,37]. To determine whether the increased WAT browning in *Pik3ca*^*H1047R*^ mice was driven by changes in hypothalamic neuropeptide expression, we quantified the levels of hypothalamic *Pomc*, *Npy* and *Agrp* in control and *Pik3ca*^*H1047R*^ mice. No change in *Pomc, Npy or Agrp* expression was observed in *Pik3ca*^*H1047R*^ mice compared to control mice, suggesting that the browning of the adipose tissue of *Pik3ca*^*H1047R*^ mice was not dependent upon stimulation of the sympathetic nervous system (Fig 6A).

To further confirm that the role of the sympathetic nervous system was not responsible for the WAT browning observed in *Pik3ca*^*H1047R*^ mice, we investigated whether sympathetic denervation would attenuate WAT browning in *Pik3ca*^*H1047R*^ mice. We injected the neurotoxin 6-hydroxydopamine (6-OHDA) unilaterally into the left inguinal fat pad of *Pik3ca*^*H1047R*^ mutant mice and monitored for changes in WAT browning 1 week later. The non-denervated (right) fat pad of *Pik3ca*^*H1047R*^ mice was used as sham control. Unilateral sympathetic denervation did not reduce WAT browning in *Pik3ca*^*H1047R*^ mice, as determined by the gross morphology (Fig 6B) and H&E (Fig 6C) of the inguinal fat pad. Denervation was assessed by tyrosine hydroxylase (TH) expression. TH is the rate-limiting enzyme in catecholamine synthesis and is a marker of sympathetic innervation [38]. WAT injected with 6-OHDA showed reduced TH staining, confirming denervation (Fig 6D). The level of UCP1 in the inguinal fat pad remained low compared to the sham control post-denervation (Fig 6D). This data suggests that WAT browning observed in *Pik3ca*^*H1047R*^ mice is driven independently of both UCP1 and sympathetic innervation. Increased WAT browning might contribute to the hypoglycaemia observed in *Pik3ca*^*H1047R*^ mice, as WAT could be taking up blood glucose to support browning.

### Ubiquitous activation of the PI3K pathway stimulates lipolysis in mice

**Fig 7:**
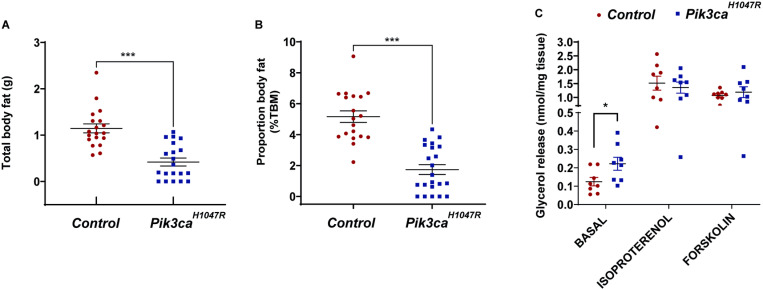
Metabolism of lipids in *Pik3ca*^*H1047R*^ mice. Control and *Pik3ca*^*H1047R*^ mutant mice were administered tamoxifen (200 mg/kg) by oral gavage on two consecutive days. Whole body fat mass, of mice (assessed 6 days post-tamoxifen) was obtained by TD-NMR and expressed as **(A)** absolute body fat (g) and **(B)** percentage of total body mass (TBM) (n = 19 controls and 22 *Pik3ca*^*H1047R*^ mice). **(C)** Glycerol release in adipose tissue, assessed in the basal state as well as in the presence of isoproterenol (1 μM) or forskolin (20 μM) (n = 8 controls and 8 *Pik3ca*^*H1047R*^ mice). Each data point represents an individual mouse and bars represent mean ± SEM. Statistical significance was determined by t-test. *** p < 0.001.

Time Domain Nuclear Magnetic Resonance (TD-NMR) analysis showed that whole body adiposity was significantly decreased in *Pik3ca*^*H1047R*^ mice compared to control mice (Fig 7A-B). Whole-body respiratory analysis demonstrated that *Pik3ca*^*H1047R*^ mice had a lower respiratory exchange rate (RER) than control mice during both the light (day) and dark (night) phases as shown above (Fig 3E), an indication that *Pik3ca*^*H1047R*^ mice oxidised more fat. Accordingly, we next examined several aspects of lipid metabolism in mice.

We examined whether *Pik3ca*^*H1047R*^ mice exhibited altered lipolysis. To do this we assessed adipose tissue *ex vivo* both in the basal state and in the presence of lipolytic agonists, isoproterenol (pan-β-adrenergic agonist) or forskolin (activator of adenylate cyclase and cAMP production independent of β-adrenergic activation). The addition of lipolytic agonists stimulated glycerol release in both control and *Pik3ca*^*H1047R*^ mice compared to the basal (unstimulated) state (Fig 7D). Most importantly, basal state lipolysis (also known as spontaneous lipolysis) was higher in mutant mice, compared to control mice, suggesting that the *Pik3ca*^*H1047R*^ mutation alone stimulates lipolysis of the adipose tissue.

The free fatty acids (FFAs) generated through lipolysis are transported into the circulation and reach other tissues such as the muscle and liver, where they can be oxidised in the mitochondria to produce ATP [39]. Therefore, we used ^14^C-labelled oleate to investigate whether there was increased FFA oxidation in the soleus (skeletal muscle) and liver of control and *Pik3ca*^*H1047R*^ mice. FFA uptake, FFA oxidation, the storage of FFAs into lipids, and the lipid storage/oxidation ratio of both the liver (Fig S1A-D) and the skeletal muscle (Fig S1E-H) were not different between genotypes.

These data show that ubiquitous expression of the *Pik3ca*^*H1047R*^ mutation reprograms adipocye lipolysis in mice towards increased production of fatty acids and glycerol, possibly to supply substrate for ATP production in peripheral tissues and glycerol for gluconeogenesis when blood glucose levels are low.

**Fig 8:**
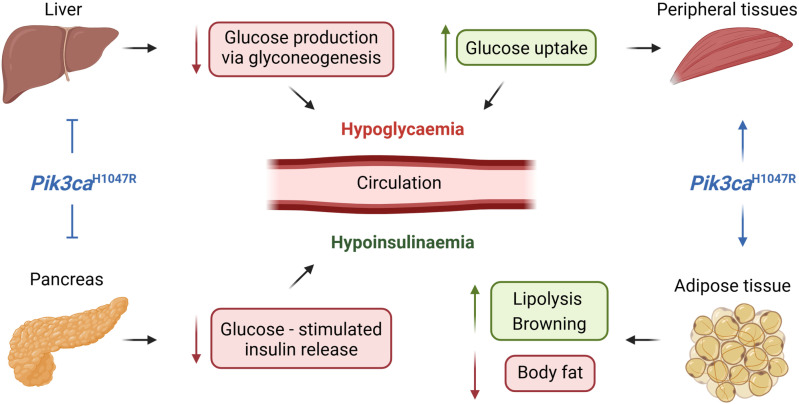
A diagrammatic summary of key findings. The ubiquitous expression of the *Pik3ca*^H1047R^ mutation leads to hypoglycaemia through a combination of the inhibition of gluconeogenesis in the liver and stimulation of glucose uptake by peripheral tissues. While a reduction in the levels of insulin in the circulation is an expected consequence of hypoglycaemia, this is further exacerbated by a direct effect of the *Pik3ca*^H1047R^ mutation in suppressing glucose-stimulated insulin release from the pancreas leading to a profound hypoinsulinaemia. *Pik3ca*^H1047R^ also induces browning of adipose tissue and an increase in lipolysis leading to a reduction in body fat. (This image was created in BioRender. Keenan, S. (2025) https://BioRender.com/x42w291).

A diagrammatic summary of the key findings is provided in Fig 8.

## Discussion

PI3K signalling is essential for the maintenance of appropriate whole-body glycaemic control, and it does so via the integration of signals from various tissues, including pancreas, liver, skeletal muscle, adipose tissue, and brain [40]. As previously reported [24], we observed that the ubiquitous expression of an activating mutation in the *Pik3ca* gene (*Pik3ca*^H1047R^) leads to severe hypoglycaemia and hypoinsulinemia, tissue growth and early death, in mice. This previous work showed that by 21 days there was an increase in the weight of most organs, including pancreas, liver, lung, and brain, that was proportional with the increase in overall body weight [24]. The exact cause of death in these mice is unclear. While our previous studies failed to find any macroscopic or histologic cause of death, we speculated that the low blood glucose levels, together with an intolerance for starvation, might suggest that the shortened life-span of these mice is likely a result of blood glucose levels falling below that required to sustain life [24].

Understanding the mechanism responsible for *Pik3ca*^H1047R^-induced hypoglycaemia is fundamental for the clinical management of patients harbouring mutations in the PI3K pathway. Defects in the PI3K/AKT/mTOR signaling pathway are considered among the genetic causes of recurrent hypoglycemia in childhood [41] and hypoglycaemia is commonly observed in patients affected by overgrowth syndromes associated with mutations in the *PIK3CA* gene [42,43].

To identify the potential mechanism responsible for *Pik3ca*^H1047R^-induced hypoglycaemia, we have explored glucose metabolism from different perspectives in our mouse model. A drop in blood glucose in mice harbouring the activating *Pik3ca*^H1047R^ mutation was not unexpected given the established role of PI3K signalling in modulating glucose uptake in insulin-sensitive tissues [44]. Indeed, treatment of patients with PI3K inhibitors leads to hyperglycaemia [45,46] presumably due to the blocking of insulin-stimulated glucose uptake. Consistent with this, we consistently observed a trend toward increased glucose uptake across most tissues in mice harbouring the activating *Pik3ca*^*H1047R*^ mutation, compared to control mice, although in retrospect our studies were likely underpowered to adequately assess this in individual tissues. Additionally, the drop in blood glucose in the *Pik3ca*^*H1047R*^ mice was compounded further by a reduction in hepatic glucose production which was associated with suppression of *Pepck* and *G6pc*, enzymes responsible for the induction of gluconeogenesis in the liver [47]. Interestingly, in patients with mosaic activating mutations in *PIK3CA,* hypoglycaemia is only observed in a subset of patients and appears to be related to the extent of mosaicism within the liver [42], an observation that is consistent with our finding of reduced hepatic glucose production in *Pik3ca*^H1047R^ mice.

Associated with the severe hypoglycaemia, we observed that *Pik3ca*^*H1047R*^ mice also presented with profound hypoinsulinemia. Since most cellular responses to insulin are mediated by PI3K [48], it is not surprising that insulin-regulated functions such as glucose uptake and suppression of hepatic gluconeogenesis are stimulated by the expression of an activating mutation in *Pik3ca*, the gene encoding the p110α catalytic subunit of PI3K, even in the absence of insulin. Of interest though, was our observation that a bolus of glucose failed to stimulate an increase in circulating insulin in the mutant mice, suggesting that expression of the *Pik3ca*^*H1047R*^ mutation in the pancreas blocks insulin release. Consistent with this, we also showed that isolated pancreatic islet cells from *Pik3ca*^H1047R^ mice failed to release insulin in response to glucose in culture. Given we have previously shown normal insulin content within the pancreas of *Pik3ca*^H1047R^ mice [24], our findings suggest that the expression of the *Pik3ca*^H1047R^ mutation in the pancreas prevents the pancreas from responding to glucose.

During exercise, glucose is taken up by the skeletal muscle through facilitated diffusion, dependent on the presence of GLUT4 in the surface membrane and an inward diffusion gradient for glucose [49]. To assess whether this process was enhanced in *Pik3ca*^H1047R^ mice, we measured the activity levels of control and *Pik3ca*^H1047R^ mice with Promethion metabolic cages. We were able to rule out exercise as a contributing factor to the hypoglycaemia in *Pik3ca*^H1047R^ mutant mice. We also did not observe any changes in food intake and efficiency ratio between control and *Pik3ca* mutant mice, therefore excluding changes in food intake as a contributing factor to the hypoglycaemia. Energy expenditure normalized to lean mass remained unchanged between control mice and *Pik3ca*^H1047R^ mutant mice, whilst the total lean body mass of *Pik3ca*^H1047R^ mutant mice increased, suggesting that energy was stored into the lean mass of *Pik3ca*^H1047R^ mutant mice to sustain lean mass growth.

*Pik3ca*^*H1047R*^ mice exhibited increased basal state lipolysis and a lower RER compared to control mice, demonstrating increased fatty acid availability and fat oxidation. This is consistent with the decrease in whole-body adiposity observed in the *Pik3ca*^*H1047R*^ mice. While this data would seem to be in disagreement with the known role of PI3K to inhibit lipolysis through inhibition of protein kinase A (PKA) [10], Duncan and colleagues have previously shown that lipolysis is stimulated during fasting [19], providing a potential explanation for the increased lipolysis observed in our hypoglyceamic mutant mice.

Interestingly, we also observed browning of WAT in the *Pik3ca*^*H1047R*^ mice. This was associated with an increased expression of genes specific for beige adipocytes, *Tmem26* and *Cd137* [50,51]*,* but not *Ucp1*. *Ucp1* is expressed at high levels in BAT under basal conditions, but is only expressed in beige adipocytes in response to activating stimuli such as exposure to cold temperature or sympathetic agonists [50]. This browning of WAT in *Pik3ca*^*H1047R*^ mice is consistent with previous studies that have shown that the insulin-PI3K axis plays an important role in the regulation of WAT browning [34,52]. The requirement for glucose in the WAT to support browning could potentially further contribute to the loss of glucose from the circulation in *Pik3ca*^*H1047R*^ mice. However, this hypothesis is yet to be tested in our model.

The lack of UCP1 expression in the WAT of *Pik3ca*^*H1047R*^ mice suggests that the *Pik3ca*^*H1047R*^ mutation regulates thermogenesis in a UCP1-independent manner. This possibility is consistent with the observation that *Ucp1* knockout mice fed a high-fat diet are resistant to the development of obesity at room temperature, suggesting the presence of a UCP1-independent pathway(s) in thermogenic adipocytes [53]. The specific mechanism underlying UCP1-independent thermogenesis in our model is not clear. However, previous studies have identified Ca^2+^ cycling and ATP-consuming futile substrate cycles as potential UCP1-independent thermogenic pathways [36,54].

In this study we have investigated the mechanism responsible for the hypoglycaemia and hypoinsulinaemia observed in *Pik3ca*^*H1047R*^ mutant mice [24]. We demonstrate that the ubiquitous expression of the *Pik3ca*^*H1047R*^ mutation induces hypoglycaemia, at least in part, by increasing glucose uptake in peripheral tissues while also inhibiting hepatic gluconeogenesis. However, it must be acknowledged that there may be other factors contributing to the hypoglycaemia that have not been explored in this study. We also show that the *Pik3ca*^*H1047R*^ mutation inhibits pancreatic release of insulin, therefore inducing hypoinsulinaemia in the mice. Finally, we report that ubiquitous expression of the *Pik3ca*^*H1047R*^ mutation stimulates fat browning independently of UCP1, suggesting the presence of alternative pathways to uncouple respiration.

## Supporting information

S1 Fig FFA oxidation in the liver and skeletal muscle of *Pik3ca*^*H1047R*^ mice. Control and *Pik3ca*^*H1047R*^ mutant mice were administered tamoxifen (200 mg/kg) by oral gavage on two consecutive days. Mice were sacrificed 8 days post tamoxifen administration and the liver and skeletal muscle harvested. FFA uptake **(A,E)**, FFA oxidation **(B,F),** FFA storage into lipids **(C,G)** and fatty acids storage to oxidation ratio **(D,H)** in the liver **(A-D)** and in the skeletal muscle **(E-H)**. For the analysis of FFAs oxidation in the liver, n = 5 control, 6 *Pik3ca*^*H1047R*^. For the analysis of FFAs oxidation in the muscle, n = 18 control, 18 *Pik3ca*^*H1047R*^. TAG = triacylglycerides, DAG = diacylglycerides. Each data point represents an individual mouse and bars represent mean ± SEM. Statistical significance was determined by t-test.
(TIF)
