# Supplementary figures and images for: Ubiquitous expression of an activating mutation in the Pik3ca gene reprograms glucose and lipid metabolism in mice
Source: PLoS One. 2025 May 12;20(5):e0322544. doi: 10.1371/journal.pone.0322544 (PMC12068571; doi:10.1371/journal.pone.0322544)

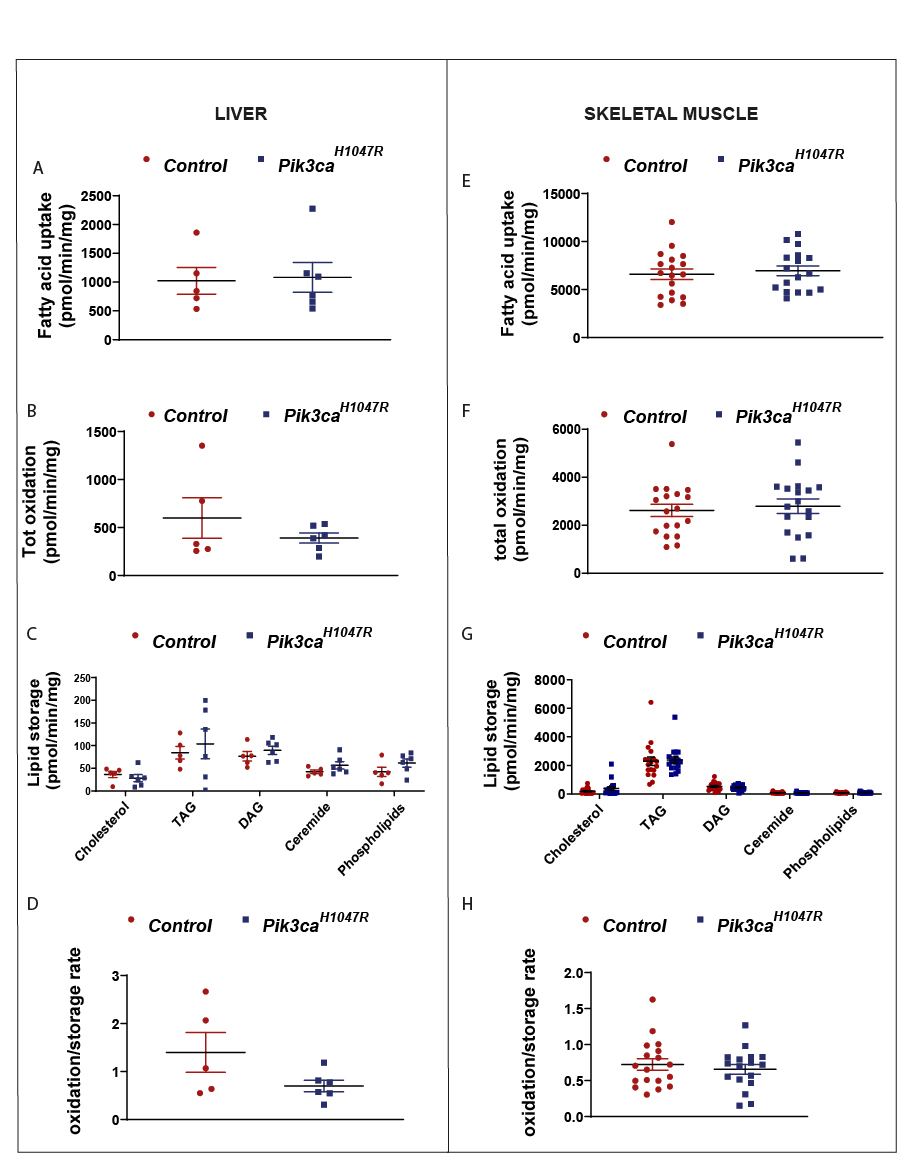

Supplement: S1 Fig — Control and Pik3caH1047R mutant mice were administered tamoxifen (200 mg/kg) by oral gavage on two consecutive days. Mice were sacrificed 8 days post tamoxifen administration and the liver and skeletal muscle harvested. FFA uptake (A,E), FFA oxidation (B,F), FFA storage into lipids (C,G) and fatty acids storage to oxidation ratio (D,H) in the liver (A-D) and in the skeletal muscle (E-H). For the analysis of FFAs oxidation in the liver, n = 5 control, 6 Pik3caH1047R. For the analysis of FFAs oxidation in the muscle, n = 18 control, 18 Pik3caH1047R. TAG = triacylglycerides, DAG = diacylglycerides. Each data point represents an individual mouse and bars represent mean ± SEM. Statistical significance was determined by t-test. (TIF) [file pone.0322544.s001.tif]
